# Calcium leaching behavior of cementitious materials in hydrochloric acid solution

**DOI:** 10.1038/s41598-018-27255-x

**Published:** 2018-06-11

**Authors:** Huashan Yang, Yujun Che, Faguang Leng

**Affiliations:** 1 School of Materials and Architecture Engineering, Guizhou Normal University, Guiyang, 550025 China; 2 China Academy of Building Research, Beijing, 100013 China

## Abstract

The calcium leaching behavior of cement paste and silica fume modified calcium hydroxide paste, exposed to hydrochloric acid solution, is reported in this paper. The kinetic of degradation was assessed by the changes of pH of hydrochloric acid solution with time. The changes of compressive strength of specimens in hydrochloric acid with time were tested. Hydration products of leached specimens were also analyzed by X-ray diffraction (XRD), differential scanning calorimetry (DSC), thermogravimetric (TG), and atomic force microscope (AFM). Tests results show that there is a dynamic equilibrium in the supply and consumption of calcium hydroxide in hydrochloric acid solution, which govern the stability of hydration products such as calcium silicate hydrate (C-S-H). The decrease of compressive strength indicates that C-S-H are decomposed due to the lower concentration of calcium hydroxide in the pore solution than the equilibrium concentration of the hydration products. Furthermore, the hydration of unhydrated clinker delayed the decomposition of C-S-H in hydrochloric acid solution due to the increase of calcium hydroxide in pore solution of cementitious materials.

## Introduction

Calcium leaching, which is a degradation mechanism consisting in a progressive dissolution of the cement hydrates as a consequence of the migration of the calcium ions to the pore solution, may severely damage structures like dams, nuclear waste containment structures, pipes, water storage tanks, and tunnels. The kinetics of calcium leaching of cementitious materials is very slow but can be a risk at long term. Predicting the long-term behaviour of cementitious materials in aggressive environment requires a sound knowledge of the various deterioration mechanisms that will affect the structures over their lifetimes. The mechanisms of calcium leaching have recently been the subject of extensive research^1–5^. Still, its physical–chemical behavior is not yet fully understood.

Amorphous calcium silicate hydrate, calcium hydroxide (portlandite), aluminates, ettringite, and unhydrated cement (clinker) etc. are the main constituents of hydrated Portland cements. Their chemical stability will strongly influence the durability of concrete in aggressive environment. The cement paste matrix is basically a porous material. These pores are generally filled with a highly basic solution. Portlandite is the most susceptible to hydrolysis because of its relatively high solubility in deionized water^6^, which is only stable when the calcium concentration in the pore solution is higher than 20 mol/m^3^. The continuous hydrolysis of portlandite exposes the cementitious constituents of the hardened cement paste to chemical decomposition. The dissolution of hydration products from hardened cement paste produces several deleterious effects, the most obvious of which are an increase in porosity and subsequent loss in strength^7–9^. Decalcification also induces dimensional changes of cement paste^10^. Berner suggested that C-S-H is stable when the calcium concentration in the pore solution is 2 to 20 mol/m^3^, such equilibrium depends on its C/S ratio (the higher the C/S ratio of the gel the higher the equilibrium)^11^. C-S-H are decalcified, reducing their C/S ratio, when the calcium concentrations below the equilibrium concentration^12^. Mineral additions to cement, such as silica fume (SF), play a significant role in the improvement of mechanical behavior and resistance to leaching^13^. Lin *et al*. reported that the resistance increased with an increase in amount of mineral additions^14^. These mineral additions decrease portandite content and form denser and more stable C-S-H than Portland C-S-H by pozzolanic effect^15^. The denser C-S-H gel decelerates the leaching process of calcium ions and slows the deterioration of strength of specimens with silica fume^16^. Also, the use of mineral additions such as silica fume is reported to result in a significant reduction of the transport propertied of the mixtures^17^. Furthermore, the calcium leaching decreases with increasing silica fume content^4^, which refine the pore structure through pozzolanic reaction, thus provide resistance to the leaching process from porosity generation^9,16^.

Leaching by deionized water is a very slow process, accelerated experimental procedures are needed. Hence, Carde introduced ammonium nitrate solution to accelerate leaching process based on the kinetics of the calcium leaching^18,19^. Calcium leaching due to ammonium nitrate solution is quicker than those obtained with the deionized water^20^. It is reported that 6 mol/L ammonium nitrate solution can accelerate the leaching speed up to 100 times while still get the same end products. Also, ammonium nitrate used is close to the condition immersed in deionized water. Therefore, ammonium nitrate was intensively used to experimentally investigate calcium leaching behavior.

Chandra^21^ investigated the process of hydrochloric acid attack on cement mortar. Tests results showed that hydrochloric acid attacks the clinker materials of Portland cement forming some soluble salts, mostly with calcium, which are subsequently leached out. This way the porosity of cement mortar is increased. De Ceukelaire^22^ studied the effect of hydrochloric acid attack on three types of mortar. It is showed that the hydrochloric acid resistance of a mortar is related best to the cement content and the formation of layers of ferric hydroxide possibly can influence the leaching processes. The hydrochloric acid also achieves an acceleration of the leaching process due to the increased calcium concentration gradient in the attacking fluid phase. Thus, the object of this paper is to investigate the extents of chemical degradation by the action of hydrochloric acid on cement paste and silica fume modified calcium hydroxide paste (SF/CH system).

## Materials and Methods

**Figure 1:**
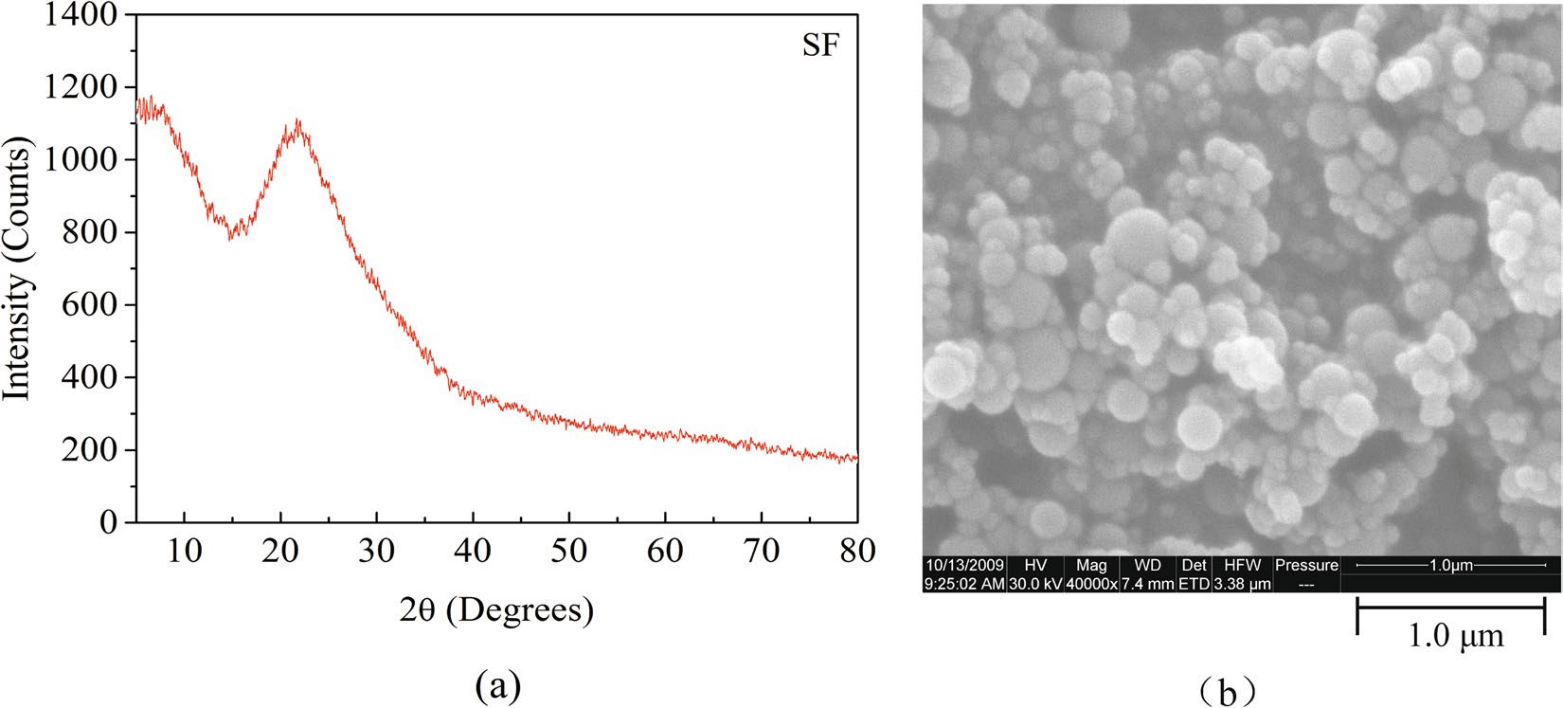
(**a**) X-ray diffractogram and (**b**) SEM image of SF.

**Figure 2:**
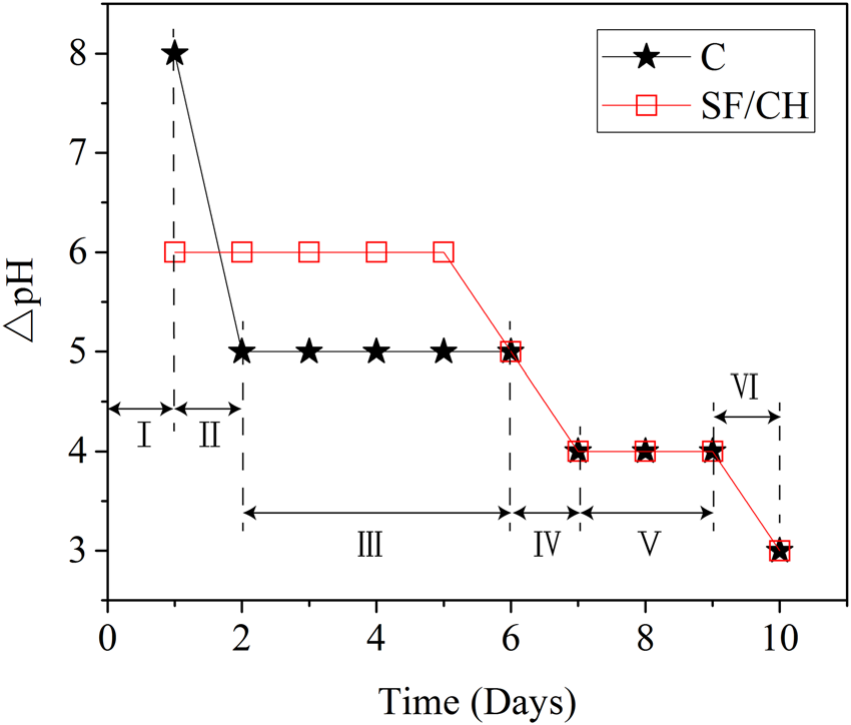
ΔpH of aggressive solutions.

Materials. The main raw materials for experimental research are moderate heat Portland cement (MHC), silica fume (SF), calcium hydroxide (CH), hydrochloric acid (HCl) and deionized water. The chemical composition and mineralogical composition of the clinker are shown in Table 1. The specific surface area of MHC is 320 m^2^/kg, and the water requirement of normal consistency is 26.8%. Calcium hydroxide and hydrochloric acid are both commercial products. It can be observed from Fig. 1 that the XRD pattern of SF shows a broad and diffuse protuberance in the vicinity of 2θ of about 25 °, indicating that SF is dominated by the glassy state. Spherical SF particles can be observed from Fig. 2, which size is less than 400 nm, the average particle size is about 160 nm.

**Table 1:** Chemical composition and mineralogical composition of investigated MHC clinker, in mass percent (data provided by cement producer).

Oxide	CaO	SiO_2_	Al_2_O_3_	MgO	SO_3_	Fe_2_O_3_	f-CaO	Ignition loss	Others
	62.2	20.9	5.1	4.3	0.6	5.7	0.5	0.3	0.4
Minerals	C_3_S	C_2_S	C_3_A	C_4_AF					
	50.0	22.1	3.8	17.4

### Methods

To study the deterioration mechanism of hardened cementitious matrix in hydrochloric acid solution, two cementitious materials were used, one was Portland cement paste and the other was silica fume modified calcium hydroxide paste (SF/CH system). It is reported that silica fume decrease portandite content and form lower C/S ratio C-S-H gel than Portland C-S-H by pozzolanic effect, thus decelerates the leaching process of calcium ions. Therefore, it is very necessary to investigate the calcium leaching behavior of SF/CH system without cement. Mix proportions of the two cementitous material were shown in Table 2. Pastes were prepared with deionized water using a water to binder ratio of 0.5. After casting in 20 mm × 20 mm × 20 mm cubic mold, specimens were demolded after 24 h and cured in saturated lime water for 28 days. After curing, the specimens were placed in hydrochloric acid solution (pH = 1) for calcium leaching. The test procedure is as follows:

**Table 2:** Mix proportions of pastes and mortar.

Type of mixes	C(%)	SF(%)	CH(%)	Water to binder ratio	Sand to binder ratio
C	100	—	—	0.5	—
SF/CH	—	30	70	1.0	—
M	100	—	—	0.5	3

Step I: After 24 h, test the pH of the solution;

Step II: Add hydrochloric acid slowly and stir with a glass rod until the pH of the solution equals 1;

Step III: Repeat steps I and II. When the pH of the solution is less than 5, sample in the middle of the specimens, and placed in anhydrous ethanol to stop hydration. Hydration products such as CH and C-S-H were analyzed by X-ray diffraction (XRD), differential scanning caloriemetry (DSC), and thermogravimetric analysis (TG).

The morphology, size and distribution of C-S-H were observed by atomic force microscopy (AFM). The atomic force microscope used in this study was a scanning probe microscope (Nanoscope IV, manufactured by Veeco) with a silicon tip. The microscope was operated in tapping mode to provide topographic maps of the cement paste using AFM techniques. During scanning the specimen was at room conditions. Different scan ranges of 1000 nm and 800 nm were used in order to better understand the surface structure and particle shapes. The results shown in this paper consist of typical results obtained from these scans. The regions used were selected after first using the optical microscope to find the most interesting regions.

Relative compressive strength of cement mortar can reflect the deterioration process of cementitious materials. Mix proportion of the cement mortar is also included in Table 2. Cement mortars were prepared with deionized water using a water to binder ratio of 0.5, sand to binder ratio of 3. After casting in 40 mm × 40 mm × 160 mm prism mold, specimens were demolded after 24 h and cured in saturated lime water for 28 and 60 days. After curing, the specimens were placed in hydrochloric acid solution (pH = 1) for calcium leaching. Control mortar were cured in saturated lime water continually until test. Adjust the pH of the hydrochloric acid solution every 24 hours until it reached to 1. After 1, 3, 5 and 20 days of leaching, the compressive strength of the specimens and control mortar were tested. The relative compressive strength of mortar is calculated according to equation (1).

1 $${\rm{Relative}}\,{\rm{compressive}}\,{\rm{strength}}=(\begin{array}{c}{\rm{Compressive}}\,{\rm{strength}}\,{\rm{of}}\,{\rm{leached}}\,\text{mortar}/\\ {\rm{Compressive}}\,{\rm{strength}}\,{\rm{of}}\,{\rm{control}}\,{\rm{mortar}}\end{array})\times 100 \% $$

### Data Availability

The data generated during the current study are available from the corresponding author on reasonable request.

## Results and Discussion

### Mechanism of calcium leaching

When hydrated cementitious materials, the pore solution of which is about 13, are in contact with hydrochloric acid, hydroxyl ions diffuse towards aggressive solution under gradient of concentration. Figure 2 shows the changes of pH of aggressive solution with time. It can be divided into six stages.

Stage I: the ΔpH of the solution of sample C was 8 (the pH of the solution was 9) and the △pH of the solution of sample SF/CH was 6 (the pH of the solution was 7). This shows that the dissolution rate of calcium hydroxide in the first stage is relatively large, which can maintain the equilibrium concentration in the pore solution of hydrated cementitious materials.

Stage II: the ΔpH of the solution of sample C decreases with time, indicating the decrease of dissolution rate of calcium hydroxide. This is because calcium hydroxide must be dissolved from the inside of the sample.

Stage III: the ΔpH of sample C was kept at 5, and the ΔpH of sample SF/CH was kept at 6, indicating that OH^−^ dissolved from the hydrated cementitious materials was just enough to neutralize H^+^ in the hydrochloric acid solution. That is, supply and consumption of calcium hydroxide reached a dynamic equilibrium.

Stage IV: the equilibrium was broken and the ΔpH of the solution of the two samples declined, indicating an increased degree of calcium leaching.

Stage V: the ΔpH of the solution declined to 4 (the pH declined to 5), indicating the second equilibrium.

Stage VI: the ΔpH of solution declined to 3 (the pH declined to 4), indicating the decreased supply of calcium hydroxide and the increased degradation of hydrated cementitious materials.

**Figure 3:**
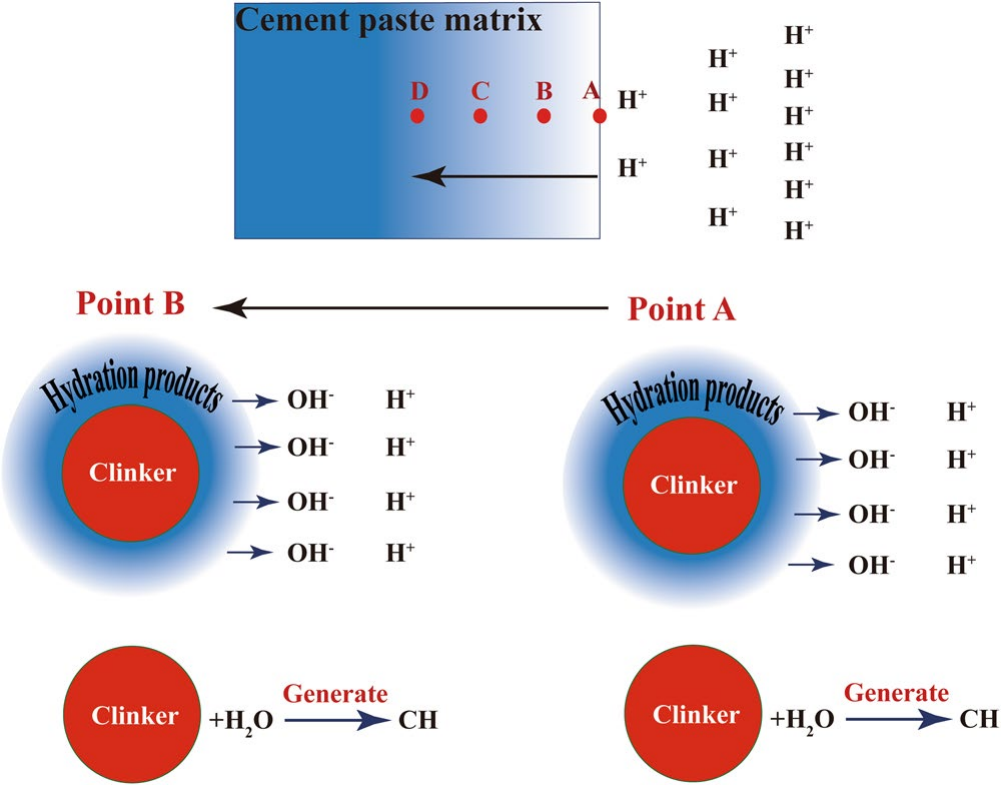
Mechanism of calcium leaching.

Figure 3 shows the degradation mechanism of hydrated cementitious materials in hydrochloric acid solution. Point A is on the surface of hydrated cementitious materials, points B, C and D are inside the hydrated cementitious materials.

The calcium hydroxide at point A is leached in the hydrochloric acid solution firstly, releasing OH^−^ to neutralize H^+^. Then, the pH in the pore solution at point A began to decrease with time. The hydration products begin to decompose when the pH reduced to the ultimate lime concentration of hydration products, resulting in an increase in porosity. On the other hand, the decrease of pH in the pore solution and the increase of porosity lead to the continuous hydration of clinker, resulting in the increase of concentration of calcium hydroxide in the pore solution again. That is, supply and consumption of calcium hydroxide is a dynamic equilibrium process, as shown in equation (2).

2 $${{\rm{V}}}_{{\rm{S}}}\rightleftharpoons {{\rm{V}}}_{{\rm{C}}},$$

Where V_S_ is the rate of supply of calcium hydroxide and V_C_ is the rate of consumption of calcium hydroxide. If V_S_ > V_C_, calcium hydroxide concentration in pore solution is above the equilibrium concentration of hydration products such as C-S-H and C-A-H, and these hydration products are stable. If V_S_ ≤ V_C_, calcium hydroxide concentration in pore solution is below the equilibrium concentration of hydration products such as C-S-H and C-A-H, and these hydration products will be decomposed. When the clinker at point A is fully hydrated, the surface of the hydrated cementitious materials is degraded. Then hydrochloric acid solution penetrates through the surface of cement paste matrix. Leaching is a combined diffusion-dissolution process where diffusion is the controlling stage^23,24^. As hydrochloric acid diffuses to point B, the calcium hydroxide at point B is leached in the hydrochloric acid solution, releasing OH^−^ to neutralize H^+^. Then, the pH in the pore solution at point B began to decrease with time. The hydration products, such as C-S-H and C-A-H, begin to decompose when the pH reduced to the ultimate lime concentration of these hydration products, resulting in an increase in porosity. On the other hand, the continuous hydration of clinker at point B results in the increase of concentration of calcium hydroxide in the pore solution again. When the clinker at point B is fully hydrated, the pH begin to reduce again. As hydrochloric acid diffuses to point C, the second equilibrium begins. In the same way, the hydrochloric acid continuously penetrates into the hydrated cementitious materials until it is completely destroyed. From the degradation mechanism of hydrated cementitious materials in the hydrochloric acid solution, it can be seen that unhydrated clinker can generate calcium hydroxide to increase the pH of pore solution. Therefore, the unhydrated clinker in hydrated cementitious materials is of great significance for the durability of concrete.

The degradation mechanism of sample SF/CH in hydrochloric acid is similar to that of cement paste. The calcium hydroxide at the surface is leached firstly, releasing OH^−^ to neutralize H^+^. Then, the pH in the pore solution began to decrease with time. The hydration products begin to decompose when the pH reduced to the ultimate lime concentration of hydration products, resulting in an increase in porosity. The hydration products will be decomposed when calcium hydroxide concentration in pore solution is below the equilibrium concentration of hydration products such as C-S-H. After that, hydrochloric acid diffuses from the surface to the inside of sample until it is completely destroyed.

### Strength of mortar

**Figure 4:**
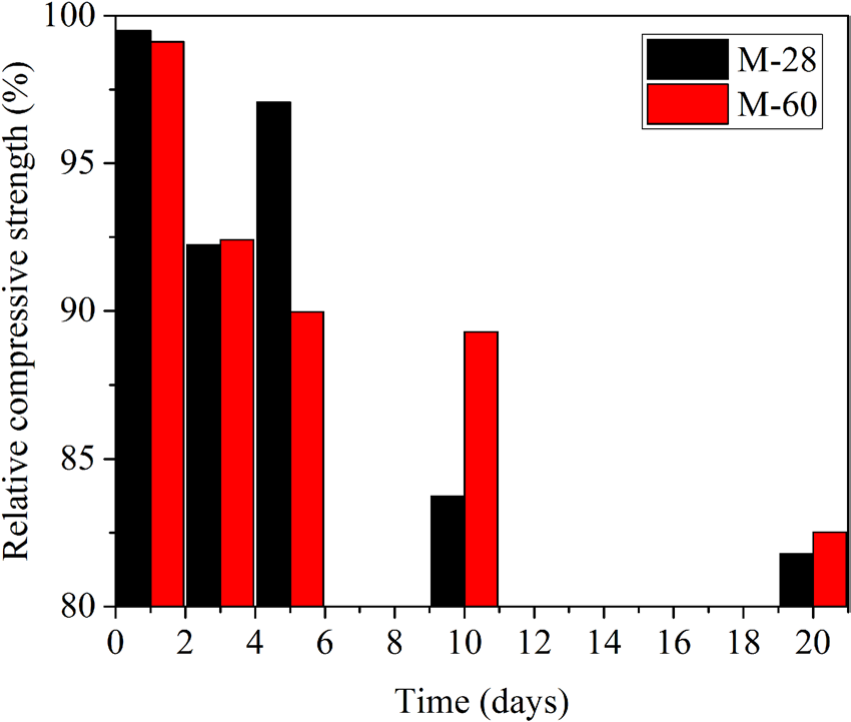
Relative compressive strength of mortar.

Figure 4 shows the relative compressive strength of sample M in hydrochloric acid solution with time. M-28 was specimen cured in saturated lime water for 28 days, and M-60 was specimen cured in saturated lime water for 60 days. It can be seen from the figure that the relative compressive strength of M-28 and M-60 slightly decreased at 1 day, which was 99% of the control sample. From the degradation mechanism of hydrated cementitious materials in hydrochloric acid solution, it can be known that the consumption rate of calcium hydroxide is below that of the supply rate, that is V_C_ ≤ V_S_. The pH of the pore solution of hydrated cementitious materials is above the equilibrium concentration of hydration products such as C-S-H and C-A-H. Therefore, hydration products such as C-S-H and C-A-H are relatively stable, and the relative compressive strength of the cement mortar decrease slightly. After that, the relative compressive strength of M-28 and M-60 tended to decrease with time. The relative compressive strength of M-28 and M-60 declined to 83% and 89% of the control mortar respectively at 10 days. This is because the consumption rate of calcium hydroxide is above that of the supply rate, that is V_C_ > V_S_, resulting in a lower pH in the pore solution of hydrated cementitious materials than equilibrium concentration of hydration products. The removal of the calcium hydroxide leads to the appearance of macro-porosity, whereas the removal of calcium in the C-S-H lead to the appearance of a micro-porosity^19,25^. The pore network in cementitious materials has great impact on their strength^26^. Therefore, the relative compressive strength of cement mortar decreases with the decomposition of hydration products. The relative compressive strength of M-28 and M-60 decreased to 81% and 82% of the control mortar respectively.

### XRD analysis

**Figure 5:**
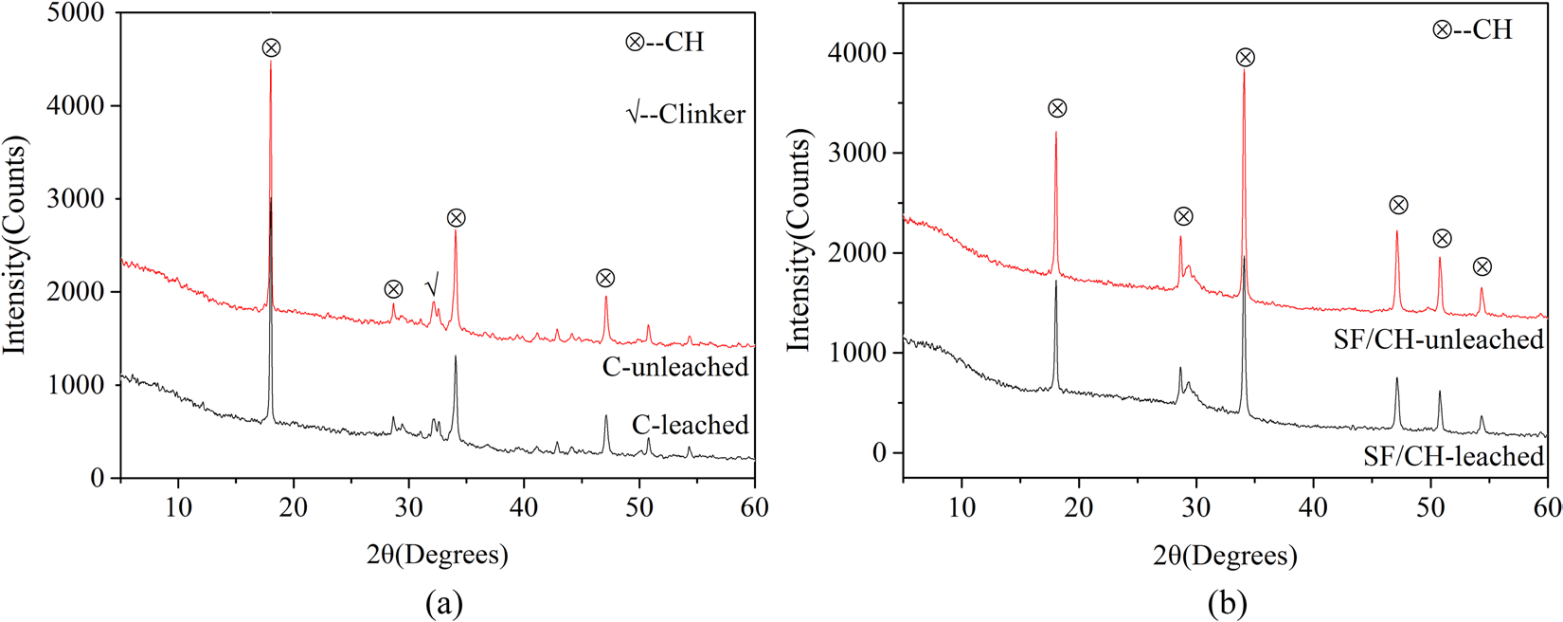
(**a**) X-ray diffractogram of C and (**b**) X-ray diffractogram of SF/CH.

Figure 5(a) shows the XRD pattern of sample C (C-leached) subjected to hydrochloric acid for 10 days. Control sample (C-unleached) cured in saturated lime water, which has the same curing time as that of the C-leached. It can be observed from the figure that the main phase of the C-leached and C-unleached are calcium hydroxide and unhydrated clinker. The diffraction peak of calcium hydroxide and unhydrated clinker in C-leached is lower than that of C-unleached. This is because part of the calcium hydroxide is leached in hydrochloric acid solution, on the other hand, the reduction of the calcium hydroxide concentration in the pore solution of hydrated cementitious materials promotes the further hydration of clinker. The hydration reaction can continuously generate calcium hydroxide to slow down the equilibrium concentration reduction in the pore solution, thereby retarding the decomposition of hydration products such as C-S-H and C-A-H. Figure 5(b) shows the XRD pattern of sample SF/CH (SF/CH-leached) subjected to hydrochloric acid for 10 days. Control sample (SF/CH-unleached) cured in saturated lime water, which has the same curing time as that of the SF/CH-leached. It can be observed from the figure that the main phase of the SF/CH-leached and SF/CH-unleached are calcium hydroxide. The diffraction peak of calcium hydroxide in SF/CH-leached is lower than that of SF/CH-unleached. This is because part of the calcium hydroxide is leached in hydrochloric acid solution. From the degradation mechanism of hydrated cementitious materials in hydrochloric acid solution, it can be known that the leaching of calcium hydroxide will lead to the decrease of pH in the pore solution of hydrated cementitious materials. When the concentration of calcium hydroxide is below that of the equilibrium concentration of hydration products, hydration products such as C-S-H and C-A-H will be decomposed.

### DSC/TG analysis

**Figure 6:**
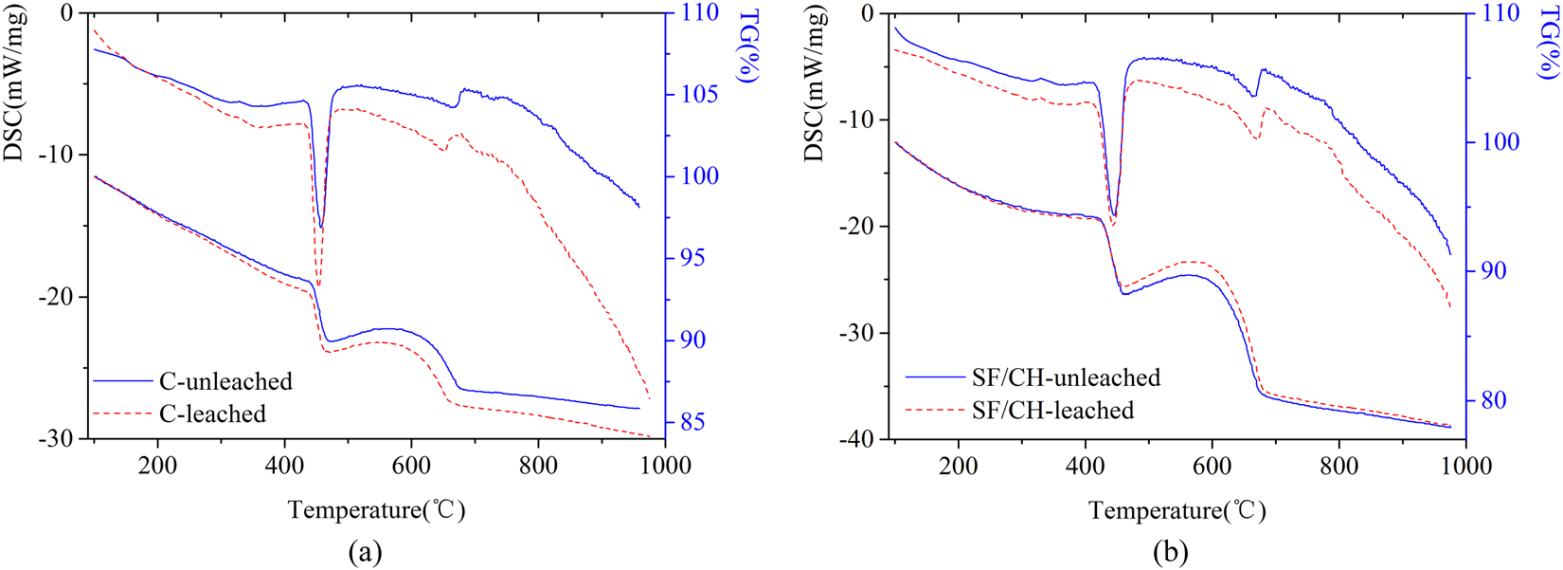
(**a**) DSC/TG curves of C and (**b**) DSC/TG curves of SF/CH.

**Figure 7:**
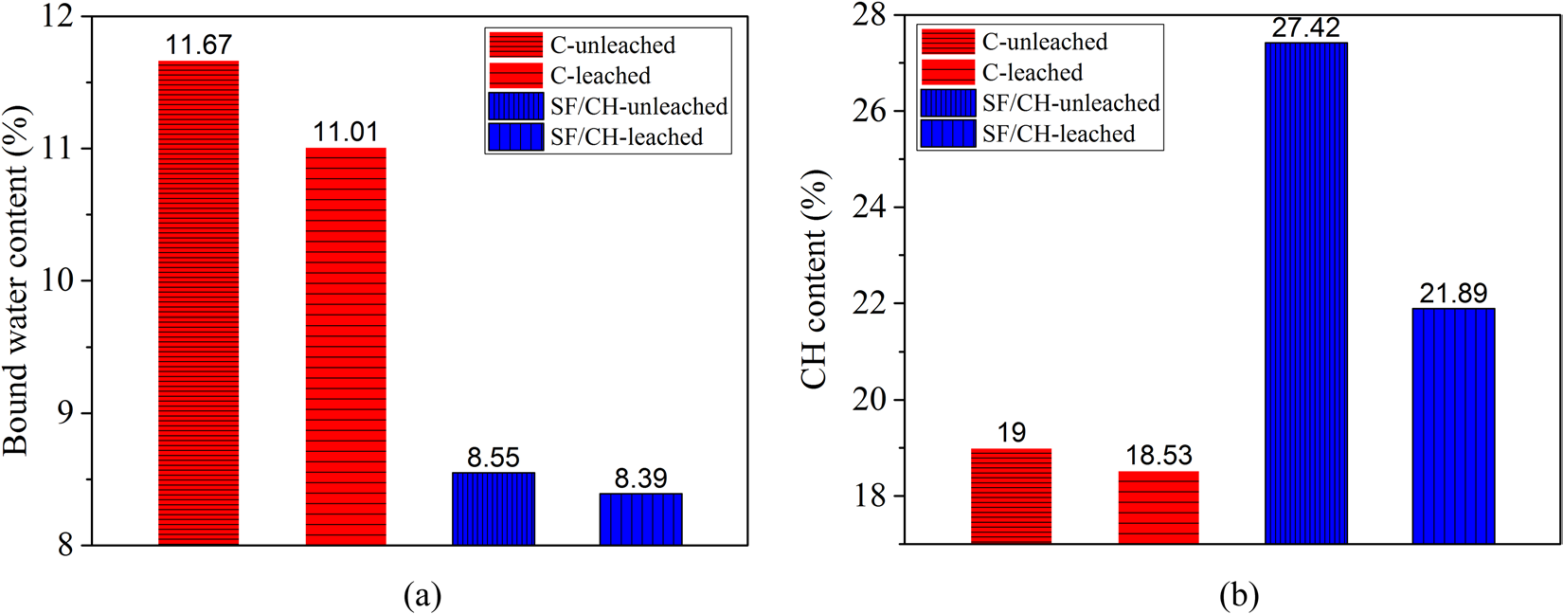
(**a**) Bound water content and (**b**) CH content.

Figure 6(a) shows the DSC-TG curves of sample C (C-leached) subjected to hydrochloric acid for 10 days. Control sample (C-unleached) cured in saturated lime water, which has the same curing time as that of the C-leached. The bound water content and calcium hydroxide content of samples were estimated from this figure. The calculation results are shown in Fig. 7(a,b).

From Fig. 6(a) and Fig. 7, it can be seen that the bound water content and calcium hydroxide content of the sample in the hydrochloric acid solution are slightly lower than that of control sample at 10 days. This indicate that the concentration of calcium hydroxide in the pore solution is lower than the equilibrium concentration, resulting the decomposition of hydration products such as C-S-H and C-A-H. Figure 6(b) shows the DSC-TG curves of sample SF/CH (SF/CH-leached) subjected to hydrochloric acid for 10 days. Control sample (SF/CH-unleached) cured in saturated lime water, which has the same curing time as that of the SF/CH-leached. The bound water content and calcium hydroxide content of samples were estimated from this figure. The calculation results are also shown in Fig. 7(a,b). From Figs 6(b) and 7, it can be seen that the bound water content and calcium hydroxide content of the sample in the hydrochloric acid solution are lower than that of control sample at 10 days. This indicate that the concentration of calcium hydroxide in the pore solution is lower than the equilibrium concentration, resulting the decomposition of hydration products. Based on the analysis results of XRD and DSC-TG, it can be seen that the calcium hydroxide was leached from hydrated cementitious materials in hydrochloric acid at 10 days. The concentration of calcium hydroxide in the pore solution decreased, and the hydration products decomposed, which led to the degradation of hydrated cementitious materials.

### AFM analysis

**Figure 8:**
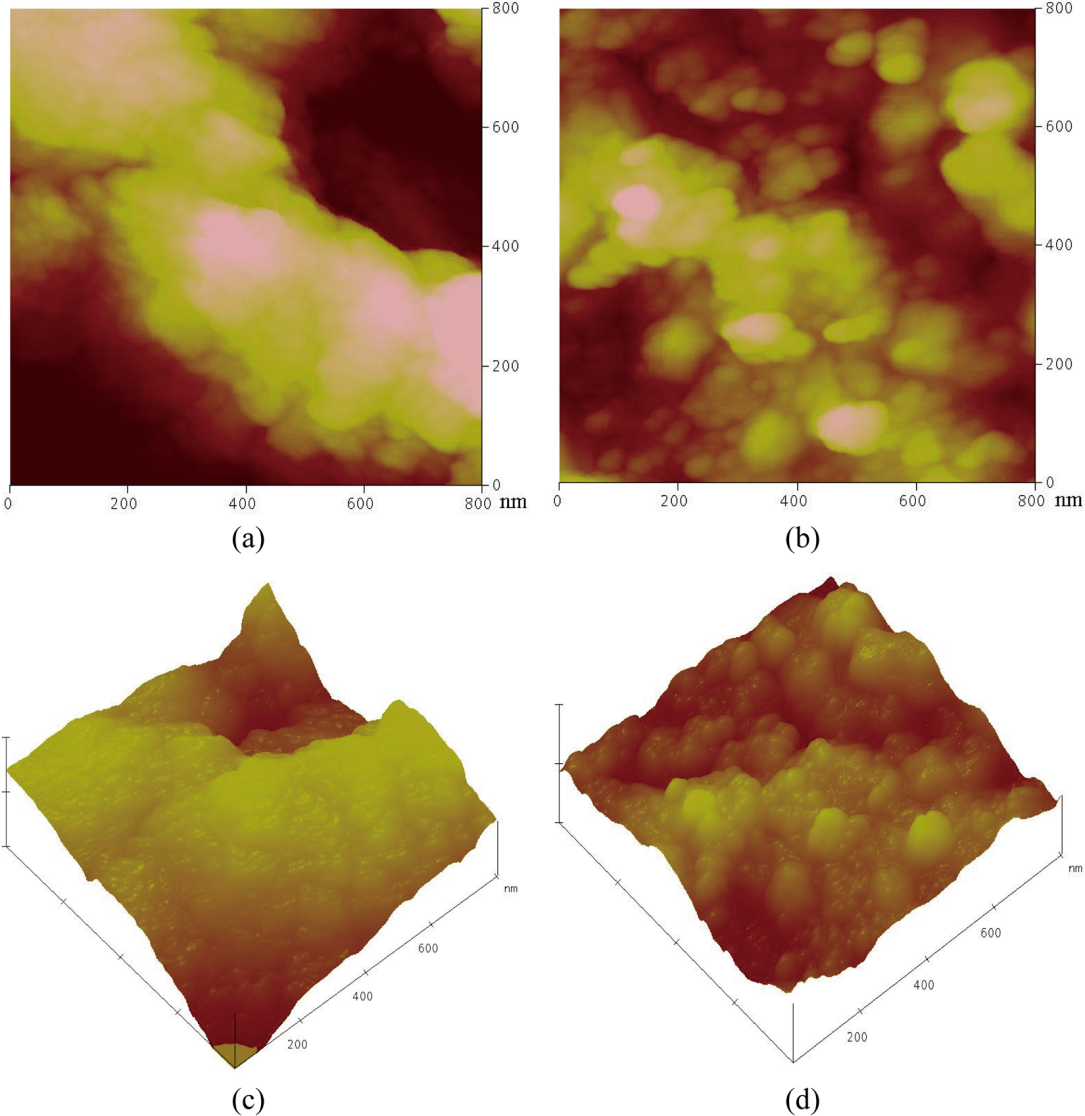
(**a**) AFM image of C-unleached; (**b**) AFM image of C-leached; (**c**) AFM 3D image of C-unleached; (**d**) AFM 3D image of C-leached.

AFM techniques was used to provide topographic maps of the selected regions, focusing on the hydration products. The color scale for the AFM images represents differences in topography, with darker colors corresponding to lower surface height and a lighter color corresponding to a higher surface height^27^. C-S-H and CH have different features, it can be clearly distinguished by the AFM technique. The CH crystals are hexagonal crystal morphology, which is a layered structure^27^. Higher magnification on the C-S-H region clearly shows this granular structure (identified by EDX, see ref.^27^), as shown in Fig. 8. Grains with sizes in the range of hundreds of nanometers are observed. It is highly noticeable that the CH region is flat and smooth and no grain structure can be observed. On the other hand, the C-S-H area is relatively rough with a grainy structure. Also, the C-S-H particles are in the range of hundreds nanometers, whereas the CH grains are in the range of dozen nm.

Figure 8(a,c) show the AFM images of sample C (C-leached) subjected to hydrochloric acid for 10 days. Control sample (C-unleached) cured in saturated lime water, which has the same curing time as that of the C-leached. Figure 8(b,d) show the AFM images of C-leached. It can be observed from the figure that C-S-H clusters of C-unleached and C-leached are made up of spherical particles with different sizes. Grains with sizes in the range of hundreds of nanometers are observed. Such a structure of the C-S-H is comparable with the description of Type III C-S-H phase as by Diamond^27,28^. The gap among C-S-H clusters in sample C-leached is larger than that of control sample (C-unleached) at 10 days. This indicates that some of the C-S-H gel in hydrated cement paste is decomposed due to the erosion of hydrochloric acid. Study shows that cohesion in cement paste results of the interactions between C-S-H surfaces in an interstitial ionic solution^29^. The interlayer calcium ions are responsible for creating attractive interparticle forces. The loss of calcium ions would also account for the continuous decrease in specific surface area of C-S-H. Thus, the loss of calcium ions of C-S-H contributes to the marked loss of compressive strength.

## Conclusions

According to the strength development and microstructure of hydrated cementitious materials in hydrochloric acid, the following conclusions can be drawn:

1. There is a dynamic equilibrium for the supply and consumption of calcium hydroxide in hydrochloric acid solution. This equilibrium moves from outside to the inside of the sample with time, thus, leading to the degradation of hydrated cementitious materials gradually.
2. The hydration of unhydrated clinker in hydrochloric acid solution can improve the concentration of the calcium hydroxide in pore solution, which delay the decomposition of hydration products such as C-S-H and C-A-H.
3. The ΔpH of the hydrochloric acid solution decreased with time, indicating the concentration of calcium hydroxide in the pore solution is lower than the equilibrium concentration, resulting the decomposition of hydration products such as C-S-H and C-A-H. The marked decrease of compressive strength observed when the pore solution of hydrated cementitious materials lower than equilibrium concentration of hydration products.
